# Re-purposing of curcumin as an anti-metastatic agent for the treatment of epithelial ovarian cancer: *in vitro* model using cancer stem cell enriched ovarian cancer spheroids

**DOI:** 10.18632/oncotarget.13413

**Published:** 2016-11-16

**Authors:** Misi He, Dong Wang, Dongling Zou, Chen Wang, Bruno Lopes-Bastos, Wen G. Jiang, John Chester, Qi Zhou, Jun Cai

**Affiliations:** ^1^ Cardiff China Medical Research Collaborative, School of Medicine, Cardiff University, Cardiff CF14 4XN, UK; ^2^ Department of Gynaecologic Oncology, Chongqing Cancer Institute, Chongqing, 400030, China; ^3^ Department of Orthopaedic surgery, Chongqing Hospital of Traditional Chinese Medicine, Chongqing, 400021, China; ^4^ Institute of Cancer and Genetics, School of Medicine, Cardiff University, Cardiff, Cardiff CF14 4XN, UK

**Keywords:** curcumin, spheroid formation, epithelial ovarian cancer (EOC), peritoneal metastasis, aldehyde dehydrogenase 1 family member A1 (ALDH1A1)

## Abstract

Malignant epithelial ovarian cancer (EOC) spheroids high frequently are detected in the malignant ascites of the patients with the extensive peritoneal metastasis of ovarian cancer, which represent a significant obstacle to efficacious treatment. Clinical data also suggested that EOC spheroids play a putative role in the development of chemoresistance. Since standard surgery and conventional chemotherapy is the only available treatment, there is an urgent need to identify a more effective therapeutic strategy. Recent studies demonstrated that curcumin exerts an anticancer effect in a variety of human cancers including ovarian cancer. This study evaluates anti-peritoneal metastasis and chemoresistance of curcumin related to the EOC spheroids. In this study, we confirm that the high invasive EOC cells forming the spheroids express a high level of a cancer stem cell (CSC) marker, aldehyde dehydrogenase 1 family member A1 (ALDH1A1), which was significantly down-regulated by curcumin treatment. Curcumin treatment markedly enhances the sensitivity of EOC spheroids to cisplatin in a dose-dependent manner. Our experiments provided evidence that curcumin could abolish the sphere-forming capacity of EOC cells in a dose-dependent manner. Moreover, curcumin substantially suppressed the growth of the pre-existed EOC spheroids, inhibited the adhesion of EOC spheroids to ECM as well as the invasion of EOC spheroids to the mesothelial monolayers. We propose to re-purpose curcumin as anti-metastatic and chemoresistant agent for EOC management in combination with conventional regimen. Further preclinical studies are necessary to validate the anti-cancer effect of curcumin in patients with EOC.

## INTRODUCTION

Epithelial ovarian carcinomas (EOCs) accounts for nearly 90% of all malignant ovarian tumours and is the leading cause of death from gynaecologic malignant tumour [1]. In contrast to most other solid tumours, the majority of EOC patients already are at the advanced stages (III or IV) disease when diagnosed. A current standard treatment for the patients with advanced ovarian cancer includes primary tumour cytoreductive surgery followed by cisplatinum-based chemotherapy since 1970 [2, 3]. Cisplatin is administrated intravenously to cause DNA crosslink, leading to apoptosis of cancer cells. Although the current therapies for EOCs have dramatically been advanced recent years, approximately 85% of patients with EOCs will have recurrent disease within 2 years and become resistant to cisplatin. Therefore, there is an urgent need to develop new therapeutic strategies for improving the efficacy of treatment of EOCs, including cisplatin.

Clinical observations suggest that there is a high incidence of the free-floating multicellular tumour spheroid superficially invading to the peritoneal cavity. In fact, ovarian cells move around readily within the peritoneal cavity via the peritoneal fluid under normal condition. Similarly, the cells shed from the primary EOCs, individually or in multicellular spheroids, are carried by the peritoneal fluid (ascites) and migrate onto the peritoneal mesothelium [4, 5], followed by sequential fast growth of tumour nodules. A study has found that the spheroid formation might enrich for cells with the cancer stem cells (CSCs)-like phenotype [6]. These CSCs can maintain an aggressive phenotype for an extended period even *in vitro* anchorage-independent culture as well as to form xenograft ovarian cancer in immune-deficient mice [7]. Failure to target tumour spheroids and eradicate the CSCs led to the development of chemoresistance or radioresistance and the disease relapse, represents a significant bottleneck to efficacious treatment [8].

Curcumin is a well-known derivative of the plant rhizome *Curcuma longa* with many pharmacological effects (i.e. anti-inflammatory, anti-oxidant, anti-bacterial and antiviral) [9]. Also, extensive studies showed that curcumin exerts cytotoxic effects on a variety of tumour types, including melanoma [10], medulloblastoma [11], breast [12], colorectal [13], pancreatic [14] and ovarian cancer [15]. Curcumin can inhibit cancer cell invasion, metastasis and angiogenesis [16] via modulating many signalling pathways (e.g., NF-κB [17], Akt/mTOR [18] and HIF1α [19]. More studies implicate that curcumin might influence the self-renewal pathways of cancer stem cells including Wnt/β-catenin [20], sonic hedgehog (SHH) [21]and Notch [22]. Nevertheless, many clinical trials reported no toxicity to human treated with curcumin at a moderate dose for several months [23].

In the present study, we generated spheroids for EOC cell lines. These high invasive sphere-forming cells express aldehyde dehydrogenase 1 family member A1 (ALDH1A1), indicating a possible enrichment of the cancer stem cells. Our data showed that curcumin could enhance the efficacy of cisplatin on ovarian cancer cells. We found that curcumin could suppress the EOC spheroid forming at dose-dependent manner with a reduction in ALDH1A1 expression. The ovarian cancer spheroids exhibit a significant increase in abilities of adhesion and invasion, which also can be inhibited by curcumin treatment. More interestingly, curcumin exerts an inhibitory effect on the ovarian cancer spheroids invading the mesothelial monolayers. This study provides evidence to re-purpose curcumin as an anti-metastatic agent for the treatment of peritoneal metastatic EOCs.

## RESULTS

### Spheroid formation of EOCs

**Figure 1:**
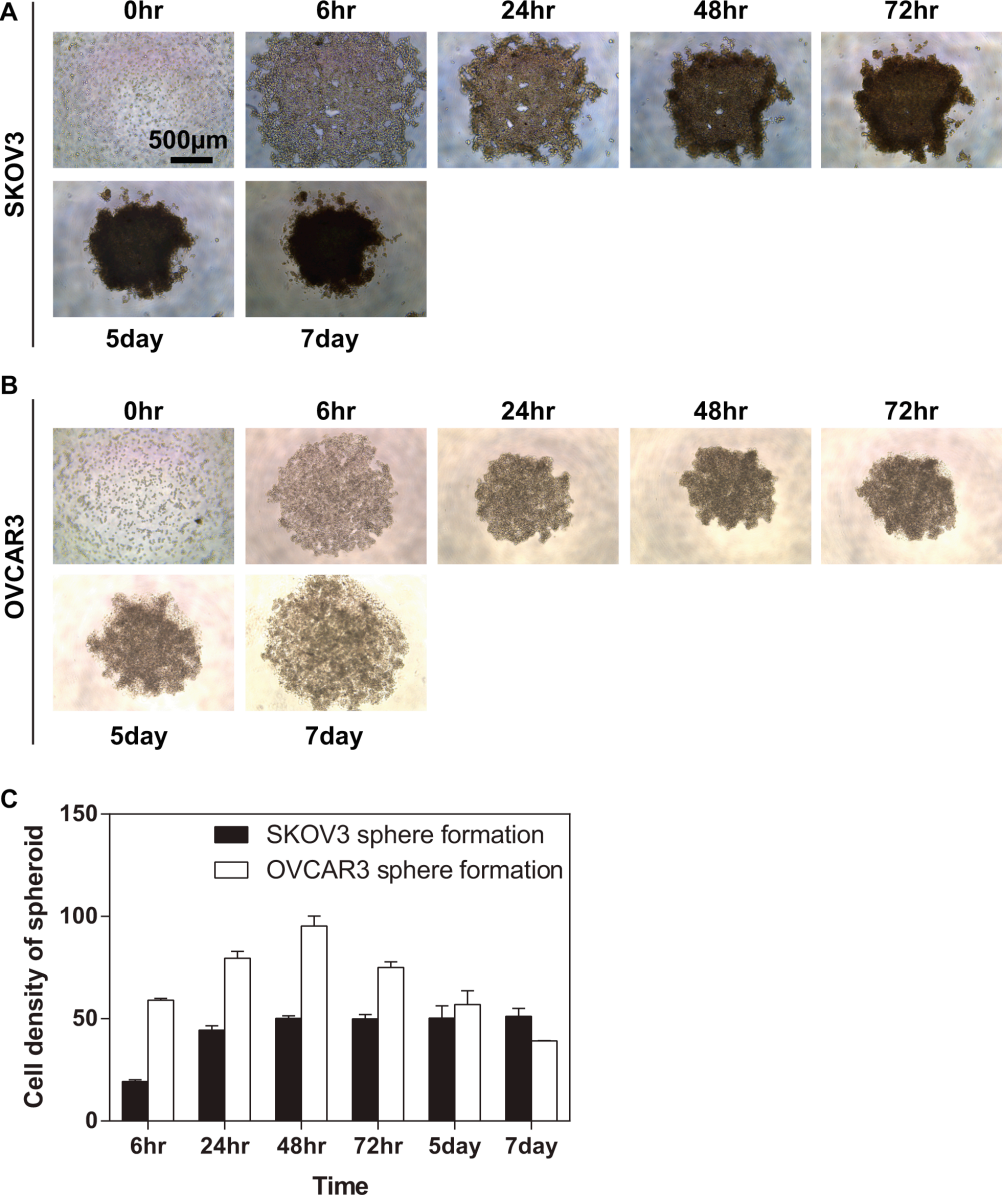
Generation and proliferation of compact spheroid from the EOC cell lines EOC cells were seeded in the ULA 96-well plate. Aggregation of cells was viewed using the light microscopy at a time course (0, 6, 24, 48 and 72 hours, 5 and 7 days). (**A**) Representative images of SKOV3 spheroids; (**B**) Representative images of OVCAR3 spheroids. Scale bar = 500 μm (**C**) Quantification of the density of EOC spheroids. The density of SKOV3 spheroids increased. By 48 hours the compaction was completed for SKOV3 cell line. Further tightening of the aggregates can be observed after two days, which could not be dissociated by pipetting. OVCAR3 cells were found to have a tendency of forming compacted spheroid much quick than SKOV3 cells. One day after plating, OVCAR3 cells formed compact spheroid which could not be dissociated by pipetting. However, after 72 hours when the cell density of SKOV3 spheroids remained a compact structure, the OVCAR3 spheroid underwent a self-dissociated process. Results were expressed as the mean ± SEM from at least three experiments.

Many different methods have been explored for tumour sphere formation, but the basic condition is to make the adhesive forces between cells greater than for the surface on the culture ware [24]. In this study, we adopted an anchorage-independent approach (described in Materials and Methods). Two ovarian cancer cell lines with different aggressiveness [SKOV3 (high invasive) and OVCAR3 (low invasive)] were trypsinised from the monolayer cultures and inoculated on an ultra-low attachment (ULA) 96-well plate. Our data showed that the two ovarian cancer cell lines exhibited different characteristics on the spheroid formation. A loose multicellular spheroid was observed in SKOV3 cells in this 3-dimensional culture condition for 6 hours, which dispersed upon pipetting, followed by a further tightening of the aggregates after two days and reaching a plateau level by day 7 (Figure 1A, 1C). In contrast, OVCAR3 cells formed the compacted spheroids much quick than SKOV3 cells (Figure 1B, 1C). However, after 72 hours, the OVCAR3 spheroid underwent a self-dissociated process (Figure 1C). Nevertheless, we successfully obtained sustainable spheroids from two ovarian cancer cell lines, which were subjected to the subsequent experiments as indicated.

### Enrichment of cancer stem cells in sphere-forming ovarian cancer cells

**Figure 2:**
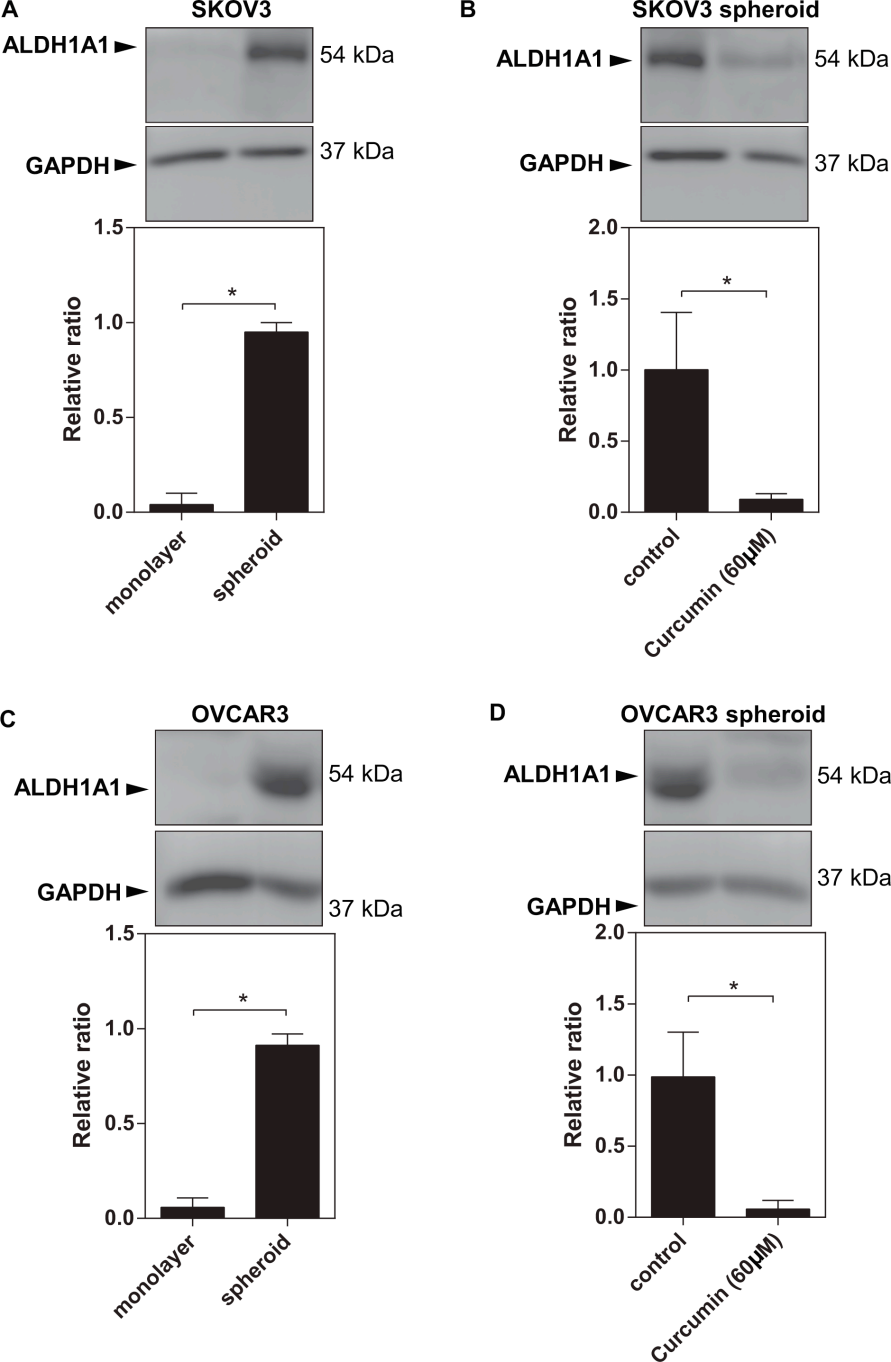
Increased ALDH1A1 expression in EOC spheroids (**A**) (**C**) Western blot data showed a high expression levels of ALDH1A1 in SKOV3 spheroids and OVCAR3 spheroids. (**B**) (**D**) Curcumin at 60 μM reduced ALDH1A1 expression in SKOV3 spheroids and OVCAR3 spheorids. GAPDH was used as an internal control. Results were expressed as the mean ± SEM from at least three experiments. *P < 0.05.

Development of ovarian cancer chemoresistance is driven by multiple mechanisms. There is an increased evidence indicating that ovarian CSC might be another crucial link for ovarian tumour cells becoming chemoresistance and metastatic. The previous study demonstrated that the spheroids formed from the surgical specimen of cancer express CSC markers depending the tumour types. In colorectal liver metastases, for instance, the spheroid forming capacity was reflected by the order of expression of group genes such as ALDH1A1^high^>CD133>CD26 [25]. Several of the characteristic CSC markers for ovarian cancer have been identified, among which high levels of ALDH1A1 are correlated with the sphere formation [26]. ALDH1A1 has been found to be up-regulated in EOC cells [27]. Since EOC cells exhibit more significant capacity of sphere-forming, we determined whether ALDH1A1 can be enriched during the EOC spheroid formation. Indeed, our Western blot analysis demonstrated that ALDH1A1 expression significantly was upregulated in the both sphered-formed EOC cells (SKOV3 and OVCAR3) compared to the monolayers of counterparts (Figure 2A and 2C).

### Cytotoxic effects of curcumin on ovarian cancer cells with sensitizing ovarian cancer spheroids to conventional therapy

**Figure 3:**
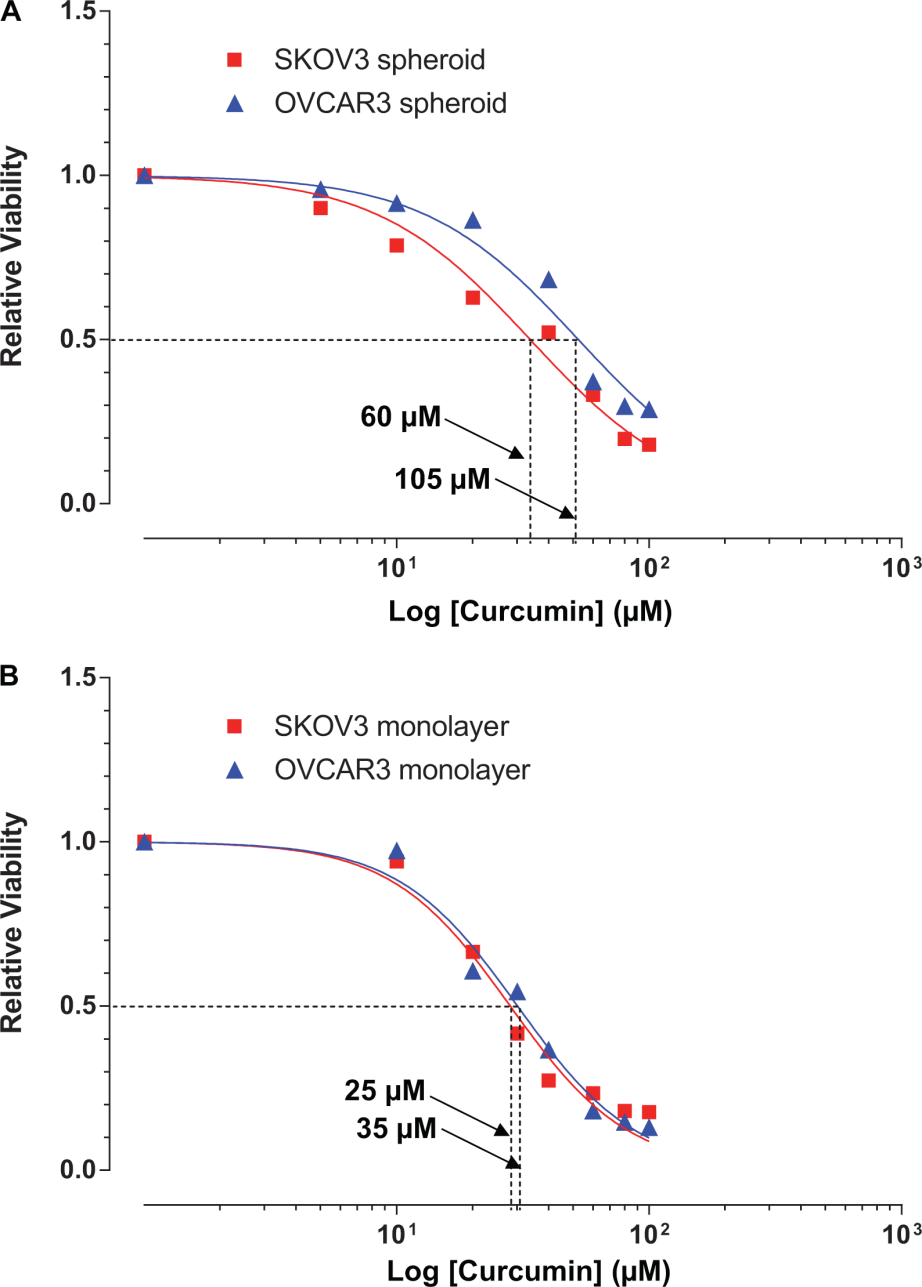
Cytotoxic effects of curcumin on SKOV3 and OVCAR3 monolayers and spheroids Cells were treated with different concentrations (as indicated) of curcumin for 48 hours. Cell viability was examined by MTT assay. Cytotoxic effects of curcumin on (**A**) EOC spheroids and (**B**) monolayer cultures. Results were expressed as the mean ± SEM from at least three experiments. *P < 0.05, compared with negative control.

Considering cell viability and cellular association are strongly interrelated, and both mechanisms might be major contributors to cancer sphere formation, we investigated whether curcumin affects the viability of the ovarian cancer cells. We treated both SKOV3 and OVCAR3 spheroids with different concentrations of curcumin for 48 hours. MTT assay showed that curcumin causes modest cytotoxicity in both SKOV3 and OVCAR3 cells from the spheroids with the IC_50_ at 60 μM and 105 μM, respectively (Figure 3A). Intriguingly, incubation with curcumin more effectively reduced viability in both SKOV3 and OVCAR3 cells under adherent monolayer cultures with the IC_50_ at 25 μM and 35 μM, respectively (Figure 3B).

**Figure 4:**
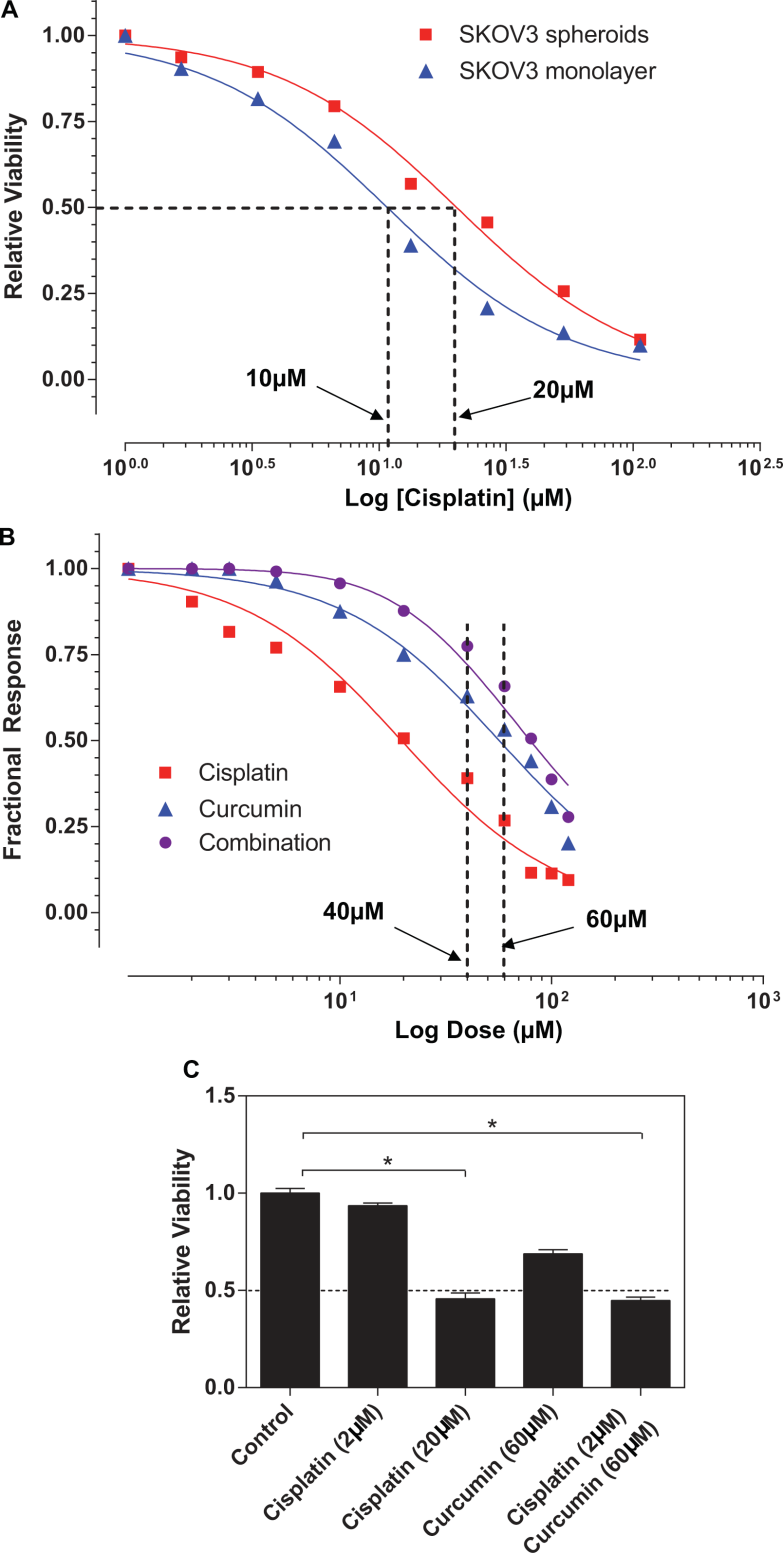
Curcumin sensitized SKOV3 spheroid to cisplatin treatment (**A**) The response to the cisplatin was examined in monolayer and spheroid by using MTT assay. Results showed represent the average in cell viability from three experiments with triplicates, ± SEM. *P < 0.05, spheroid vs. monolayer. (**B**) Bliss independence models for cisplatin and curcumin interaction indicated that additive model was observed at the lower range of drug concentrations (< 40 μM or > 60 μM), whereas the combination seems to display Bliss synergism at concentrations (of 40 μM and 60 μM). (**C**) The combined with curcumin (60 μM), 0.5 μg/ml of cisplatin (equivalent to 3.33 μM) exerted ∼50% of inhibitory effects on SKOV3 spheroids (which can be only achieved by cisplatin alone at 16 μg/ml) and combination of these two agents. Results were expressed as the mean ± SEM from at least three experiments. *P < 0.05, compared with negative control.

Meantime, we found that there is a difference in the inhibitory effects of cisplatin on the SKOV3 cells between in monolayer cultures and spheroids. To determine whether the spheroid formation of ovarian cancer cells conferred their chemoresistance, we treated both the SKOV3 cell monolayers and spheroids with cisplatin for 48 hours. As shown in Figure 4A, the SKOV3 spheroids require a higher concentration of cisplatin (6 μg/ml≈ 20 μM) to achieve the 50% inhibition of cell growth than the SKOV3 in monolayer culture (3 μg/ml≈10 μM).

Given that cisplatin and curcumin have shown some degrees of toxicities to the SKOV3 spheroids, it is logical for us to assess what combine effects of cisplatin and curcumin treatment on SKOV3 spheroids. Of particular interest is to identify whether synergism when both drugs tested in combination. We adopted for Bliss independence models for drug interaction. As shown in Figure 4B, additive model was observed at the range (< 40 μM, > 60 μM) of drug concentrations, whereas the combination seems to display Bliss synergism at 40 and 60 μM. Our data further demonstrated that combined with curcumin (60 μM), 2 μM of cisplatin (equivalent to 0.5 μg/ml) exerted ∼50% of inhibitory effects on SKOV3 spheroids (which can be only achieved by cisplatin alone at 20 μM≈6 μg/ml) (Figure 4C), suggesting that the combination of curcumin might sensitize ovarian spheroids to conventional chemotherapy and a significant reduction in dose of conventional chemoagents.

### Inhibitory effects of curcumin on spheroid formation of ovarian cancer cells

**Figure 5:**
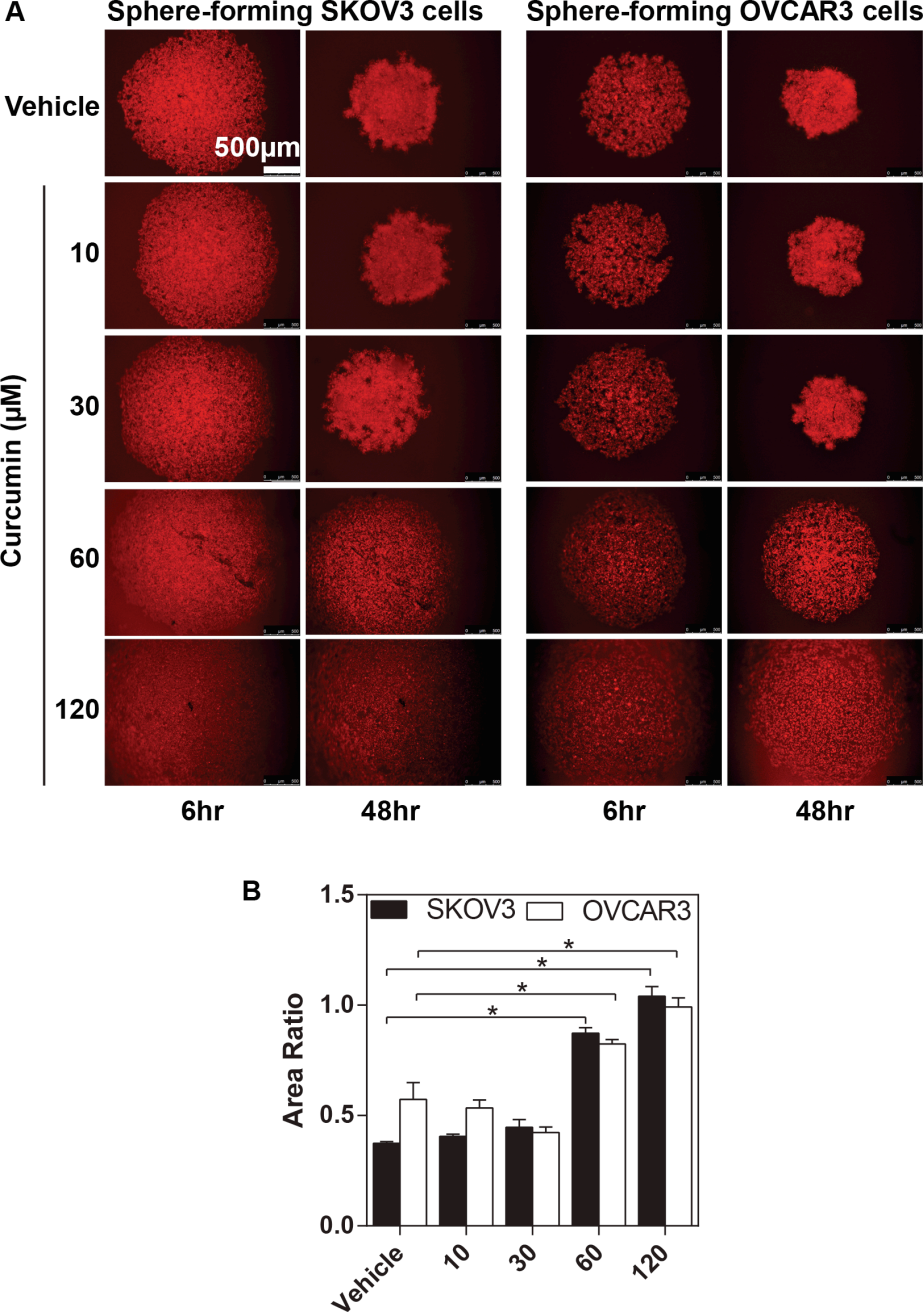
The inhibitory effects of curcumin on SKOV3 and OVCAR3 sphere formation The SKOV3 and OVCAR3 cells formed spheroid in different concentrations (as indicated) of curcumin for 48 hours. The process of sphere formation was observed at 6 hours and 48 hours. (**A**) Representative images of sphere-forming SKOV3 (the left-hand panel) and OVCAR3 cells (the right-hand panel). Calibrated images were used to obtain morphometric data to determine spheroid area. (**B**) The changes of EOC spheroid area were normalized to spheroid area at 6 hours. Results were expressed as the mean ± SEM of at least experiments. *P < 0.05, compared with curcumin 0 μM. Scale bar = 500 μm.

To investigate whether curcumin affects the sphere-forming of ovarian cancer, we examined the fluorescent densities of EOC spheroids formation for 2 days in culture. The fluorescent staining shows an intensive rim area of SKOV3 spheroid with a less densely middle section (the top left-hand panel at Figure 5A), whereas OVCAR3 cells exhibited more even intensity over the spheroids, (the top right-hand panel at Figure 5A). We found that curcumin exhibits an inhibitory effect on the compaction of the ovarian cancer spheroids at a dose-dependent manner (Figure 5B). Curcumin completely impairs the round geometry of the tighten spheroids at 60 μM for SKOV3 and at 120 μM for OVCAR3 (Figure 5B).

Since curcumin is a potent inhibitor of the ovarian cancer sphere formation, we performed western blot analysis to determine whether curcumin can modulate ALDH1A1 expression in ovarian cancer spheroids. Curcumin treatment (60 μM) resulted in a significant reduction in ALDH1A1 expression with completely disrupting the sphere formation of EOC cells (Figure 2B and 2D). These data confirm that curcumin might act as a promising agent targeting the cancer stem cells.

### Curcumin inhibits the EOC spheroids adhering and invading to the extracellular matrices (ECM)

**Figure 6:**
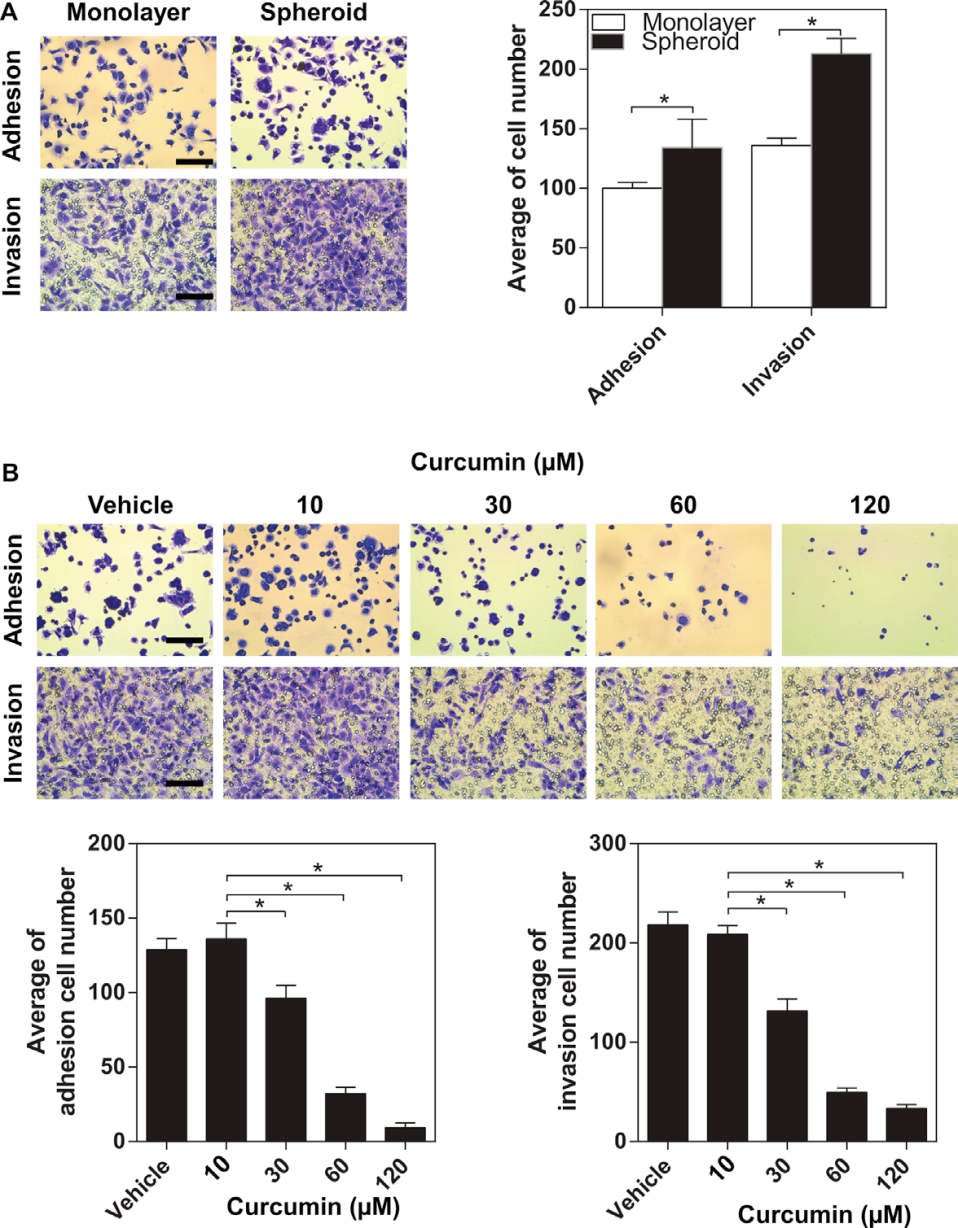
The inhibitory effect of curcumin on aggressive capacity of SKOV3 spheroid (**A**) Comparison of adhesion and invasion of SKOV3 between monolayer and spheroid. Cells grown as spheroid for 2 days were trypsinized to a single cell suspension and allowed to adhere or invade to the matrigel. *P < 0.05, compared with monolayer. (**B**) Adhesion and invasion assays were performed in SKOV3 spheroid after curcumin treatment. Scale bar =125 μm. All the data were presented as the mean ± SEM of at least three experiments.*P < 0.05, compared with negative control.

Progression of ovarian cancer cells involves adhesion and interaction with the extracellular matrix of the surrounding tissues. In this study, we used adhesion and invasion assays, in which cells need to adhere or break down the Matrigel mimicking the cells interactive with the extracellular matrix (ECM) *in vivo*. We analysed the adhesion and invasion assay for malignant EOC cell line-SKOV3 cells (either in monolayer or spheroid cultures). SKOV3 spheroids exhibited significantly adhesive to the Matrigel (*p* < 0.05) after being added for 45 minutes compared with SKOV3 in the monolayer culture (Figure 6A). For the invasion assay, the SKOV3 cells both in monolayer culture and spheroids were added onto the Matrigel-coated membranes (inserts) for 48 hours. Similarly, compared the SKOV3 cells in monolayer culture, the SKVO3 spheroids exhibited significantly increased ability to invade across the Matrigel membrane (*p* < 0.05) (Figure 6A), These data indicated that ovarian cancer cells obtained more aggressive properties after forming spheroids, supporting the notion of the increased metastatic attributes of a tumour spheroid.

We assessed whether curcumin exert anti-adhesion/invasion effects on ovarian cancer spheroids. The tested doses of curcumin were 30, 60 and 120 μM. After treated with curcumin for 48 hours, a dose-dependent reduction in the adhesion of SKOV3 spheroids to the Matrigel was observed and the reduction was highest at 120 μM (Figure 6B). As shown in Figure 4B, curcumin reduced the invasion ability of SKOV3 spheroids dose-dependently, with a maximum decrease ∼90% (*p* < 0.05) at 120 μM. The data showed that curcumin significantly decreased spheroid-ECM adhesive and invasive capacities at a dose-dependent manner (Figure 6B).

### Inhibitory effect of curcumin on EOC spheroids invading the mesothelial monolayers

**Figure 7:**
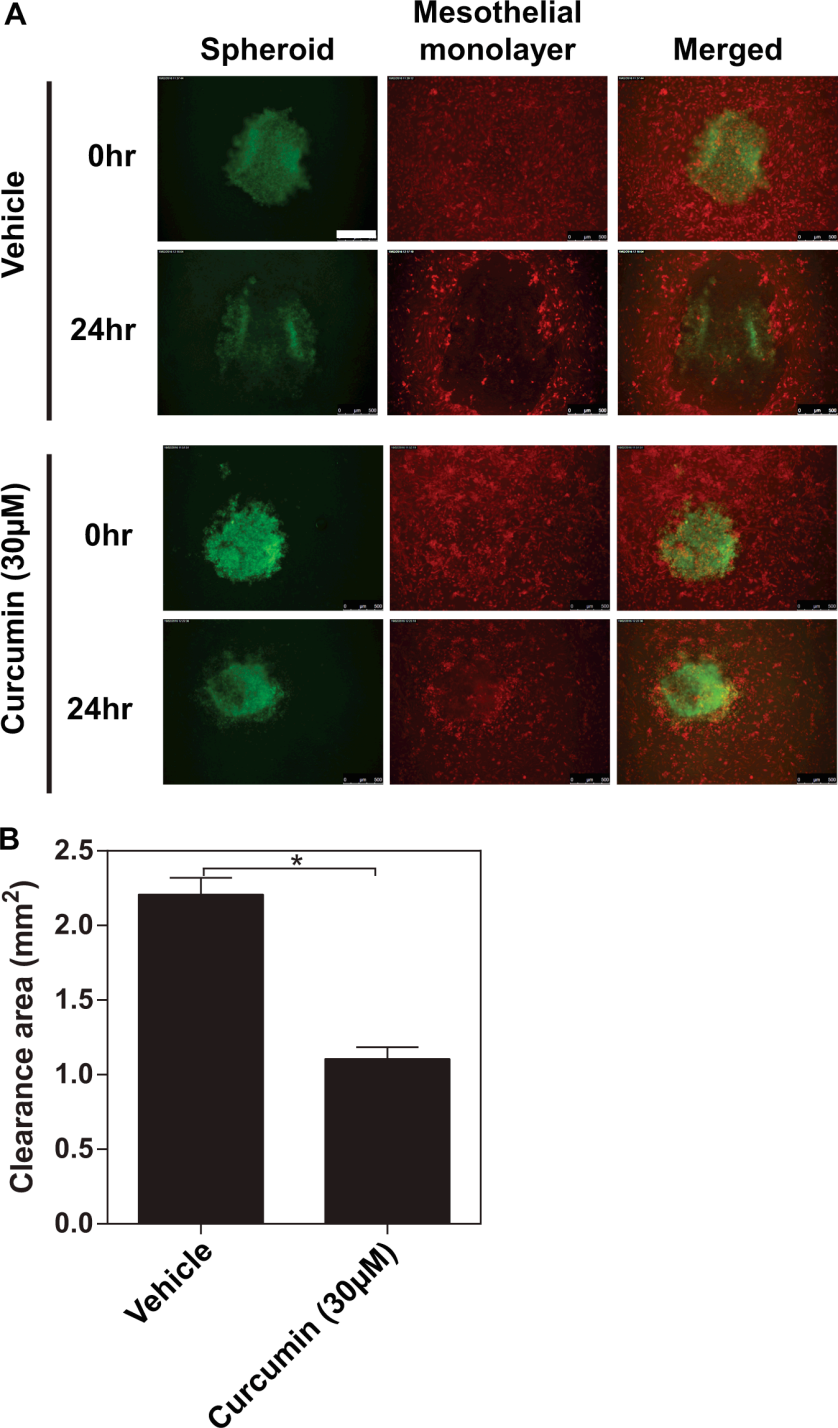
The inhibitory effects of curcumin on ovarian cancer spheroids invading the mesothelial monolayer Followed by adhering, the invading edges of the spheroids push the mesothelial cells aside clearing off an acellular area in the monolayer. (**A**) Representative images of immunofluorescence for SKOV3 spheroid (Green) invading mesothelial monolayer (Red) for 24 hours. (**B**) Quantification of the clearance areas on mesothelial monolayers by curcumin. All the data were presented as the mean ± SEM of at least three experiments. *P < 0.05, compared with negative control. Scale bar = 500 μm.

Since all of the organs within the peritoneal cavity are covered with a continuous single layer of mesothelial cells, a critical step for the peritoneal cancer metastasis is for cancer cells to sustain proliferative and invasive abilities after adhered to the mesothelium. In the attempt to better understand whether curcumin affects the invasive behaviour of EOC spheroids onto the mesothelial cells of peritoneal cavity, we directly analyzed SKOV3 spheroids interactions with human mesothelial cell (LP-9) monolayers. LP-9 cells were labelled with CellTracker^™^ Orange CMRA (red) while the SKOV3 spheroids were labelled with CellTracker^™^ Oregon Green 488 (green). Followed by adhering, the invading edges of the spheroids push the mesothelial cells aside clearing off an acellular area in the monolayer. The resultant area, then, can be measured as mesothelial clearance. After 24 hours of co-cultured with the SKOV3 spheroids, the LP-9 monolayers exhibited a considerable size of acellular area compared to the original size of the spheroids (Figure 7A, the top two panels). As shown in Figure 7A (the bottom two panels), there were much smaller acellular area created by the SKOV3 spheroids pre-treated with curcumin (30 μM for 48 hours). As Figure 7B shown, curcumin may exert a significant inhibitory effect on the mesothelial invasiveness of ovarian cancer spheroids.

## DISCUSSION

EOCs are responsible for approximately 90% of all ovarian cancer cases and derived from ovarian surface epithelium stem cells or fallopian tube epithelium. Despite decades of medical advances, ovarian cancer is still associated with high number of patients having a relapse of their disease. Strong evidence suggests that a rare subpopulation of cancer cells called ‘cancer stem cells’ (CSCs) is responsible for the escaping the current chemotherapy and tumour recurrence. Therefore, targeting CSCs represents an important strategy to greatly improve current cancer treatment.

The accumulated data suggest that cancer sphere-formation is able to enrich dominant self-renewal of the CSC population. It has been demonstrated that only cells forming spheroids in anchorage-independent conditions, but not the adherent cells, could be returned to exponential growth under normal culture conditions and then back to spheroid conditions to reform spheroids [6]. In order to study EOC tumorigenesis, we generated self-renewing spheroids when EOC lines were grown in anchorage-independent conditions. These spheroids morphologically are resemble to spheroids isolated from patient ascites. Although the resultant spheroids enriched of CSCs are heterogeneous, these spheroids could restore to exponential growth under normal adherent culture condition, and then much easily form spheroids when returned to anchorage-independent conditions (Data no shown). Extensive studies revealed that hypoxia, nutrient deprivation, abnormal distribution of metabolites and cell-cell interactional signalling molecules is difficulty to be recapitulated in two-dimensional (2D) monolayer of cancer cell cultures. In contrast, three-dimensional (3D) structures provide a spatial and temporal insights on how cancer cells exposure to heterogeneous oxygen and nutrients, but also mimic a chemoagent gradient [28]. Nevertheless, cellular behaviour and treatment response comparisons of CSC and non-CSCs often have to be made between cells propagated in different conditions (spheroids versus adherent growth). Many studies have successfully identified ovarian CSCs based on the expression of stemness markers. For instance, aldehyde dehydrogenase 1 family member A1 (ALDH1A1) ALDH1A1 has been exploited to define CSC subpopulations in ovarian cancer [27]. ALDH1A1 positive cells isolated from ovarian cancer cell lines are chemoresistant and significantly more tumorigenic capacity than ALDH1A1 negative cells. However, these ovarian CSC markers seem not to be good candidates for CSC-targeting due to these differential expressed cell surface markers having not been functionally linked with ovarian CSC properties such as self-renewal or differentiation [29].

The current model of EOC peritoneal metastasis includes three steps. Firstly, cells need to leave from the primary tumour. Then, cells are transiting in the peritoneal cavity. Although both single cell and spheroid cells are contributed to the metastasis, it has been proposed that spheroid possesses increased invasive ability [7]. The final step is invaded the mesothelial lining and grow as a second lesion [30, 31]. We speculated that our sphere-formation approach might also have advantage of dissecting ovarian cancer peritoneal metastasis. A recent report has shown highly correlation between a selective increase in CSCs spheroids and peritoneal metastasis [32]. Also, cancer spheroid cells are well known to have a great tendency to tolerate many chemoagents than their monolayer counterparts. In general, these spheroids possess rough-rounded morphology and the capable of keeping intact as free-floating conditions. Since it is believed that the established ovarian cancer cell line do not necessarily reflect *in vivo* conditions, many sophisticated heterotypic ovarian cancer spheroids containing stromal cells have been reported. However, co-cultured with different types of cells the ovarian cancer cells are difficult to form spheroids with standardized mass production [33]. Nevertheless, we observed that the single ovarian cancer cell-formed spheroids can lead to reproducible sphere formation within similar size, which makes these spheroids ideal for drug testing.

**Figure 8:**
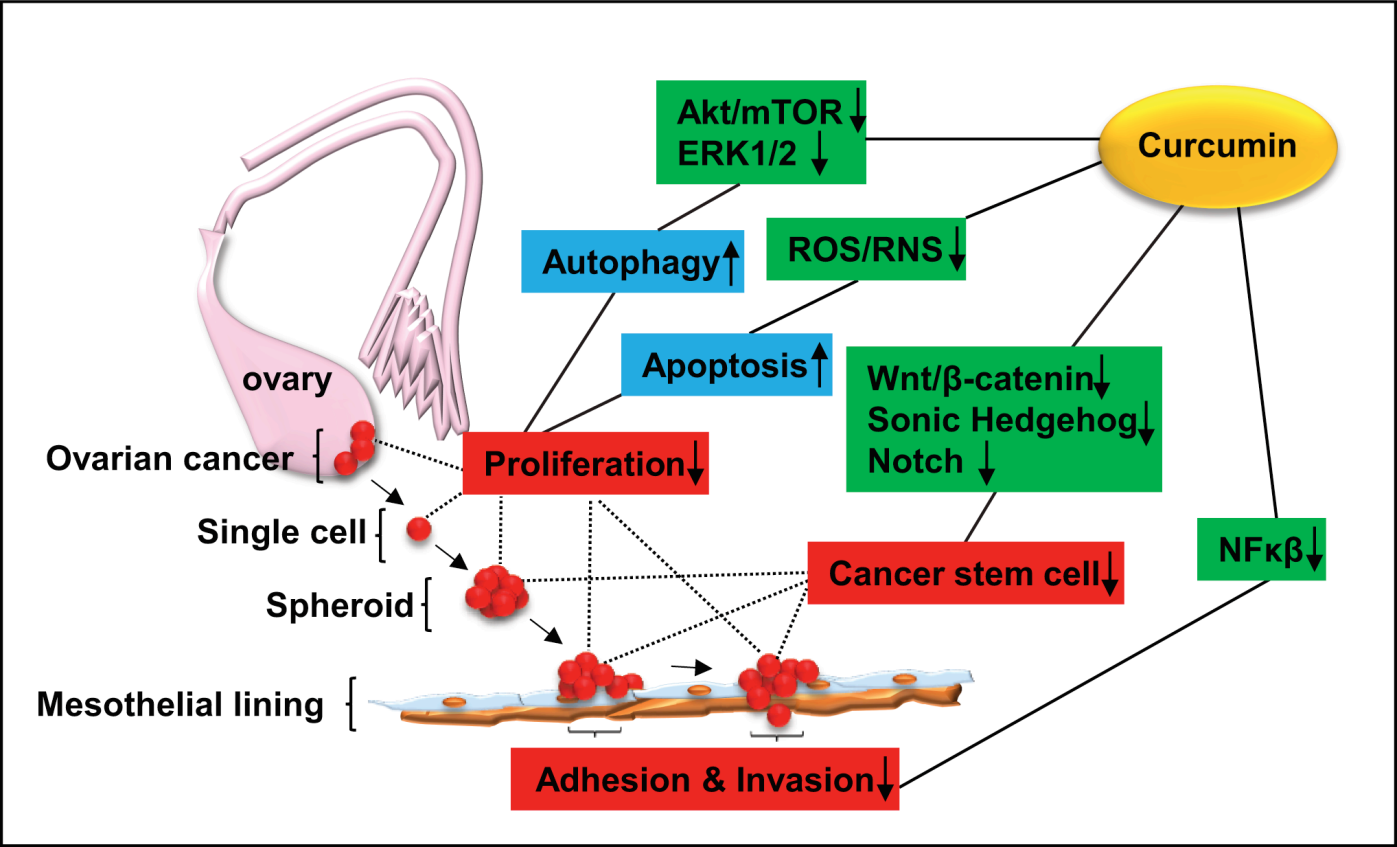
Schematic diagram showing the possible signalling pathways related to the inhibitory effect of curcumin on ovarian cancer development Curcumin inhibits the activities of Akt/mTOR, ERK1/1, leading to induction of autophagy, while ROS production is rendered by curcumin, resulting in activation of apoptotic signaling [18]. Taken together, curcumin exerts inhibition of ovarian cancer cell proliferation. Curcumin reduces the stemness of ovarian cancer cells through downregulation of Wnt/β-catenin [20], Sonic Hedgehog [21] and Notch pathways [22]. Curcumin inhibits the adhesion and invasion of ovarian cancer cells to peritoneal mesothelial lines via inhibition of NFκβ signaling activation [17]. Akt (pathway): a serine/threonine kinase involving cell survival and growth; ROS/RNS: Reactive Oxygen Species and Reactive Nitrogen Species; Wnt/β-catenin: a group of signal transduction pathways associated with tumour development; NFκβ: nuclear factor κ beta.

Despite numerous compounds have shown pre-clinical promise as new ovarian cancer therapeutic agents no drugs have successfully improved the overall survival for the patients with ovarian cancer during the past 30 years [34–36]. Addition to the lack of response, chemoresistance may result in use of high-dose chemoagents, leading to unnecessary toxicities to normal host tissues/cells, some of which are even fatal. Thus, the identification of nontoxic agents that specifically target the CSC enriched ovarian cancer spheroids may serve as a novel therapeutic avenue to improve the efficacy of current chemotherapeutic agents, which in turn leading to reducing the risk of relapse and peritoneal metastasis. Curcumin is extensively used as a flavouring agent in food supplements and is responsible for giving turmeric spice yellow colour. Curcumin directly interacts with several molecular proteins including inflammatory molecules, cell survival proteins, histone acetyltransferase (HATs), histone deacetylase (HDAC), protein kinase and reductase, glyoxalase (GLOI), proteasome, sarcoplasmic reticulum Ca2+ ATPase (SERCA), human immunodeficiency virus type 1 (HIV1) integrase and protease, DNA mehtyltransferases 1 (DNMT1), FtsZ protofilaments, carrier proteins, DNA, RNA, and metal ions [37]. Curcumin also interacts indirectly with several transcription factors including nuclear factor-kappa-B (NF-κB), activator protein 1 (AP-1), β-catenin, signal transducer and activator of transcription (STAT) protein, and peroxisome proliferator-activated receptor y (PPARy) (Figure 8). Recently, it has become clear that curcumin, the major derivate of turmeric, has no detectable toxicities [23], but still induces chemo-/radio-sensitization in ovarian cancer cells by enhancing their apoptosis [38]. Our study confirmed that curcumin treatment resulted in an enhancement in inhibitory effect of cisplatin on the viability of the ovarian cancer cells. Furthermore, we demonstrated a novel inhibitory effect of curcumin on ovarian cancer cells through affecting the ovarian cancer spheroids. Recent studies demonstrate that curcumin affects the extracellular matrix (ECM), adhesion molecules via regulation of PI3K/Akt, ERK and PTEN signalling pathway [39] (Figure 8). Using a single ovarian cancer cell-forming multicellular spheroids, we revealed that curcumin induced the inhibition of sphere formation corresponded with downregulation of proliferation, adhesion to and/ or invasion through the extracellular matrix (ECM). Most importantly for the first time curcumin was shown to attenuate ALDH1A1 expression in the EOC spheroids. Moreover, we observed that curcumin decreased the mesothelial clearance ability of the spheroid, suggesting that curcumin prevents ovarian cancer cells invasion at metastatic site.

In conclusion, we confirmed that the sphere formation plays a vital role in peritoneal metastasis and chemoresistance in epithelial ovarian cancer. We highlighted the therapeutic value of intervening the ovarian cancer spheroids in the inhibition of peritoneal metastasis. Targeting both the sphere formation and cancer stemness might be another promising strategy in overcoming chemoresistance in ovarian cancer management. Collectively, our results provide clear evidence of the therapeutic potential of curcumin as an adjunct to conventional chemotherapy in the ovarian cancer patients with peritoneal metastasis and/or being resistance to conventional chemoagents. Our findings strongly support the effort to re-purpose curcumin as anti-metastatic therapeutic agent for EOC.

## MATERIALS AND METHODS

### Spheroid formation of EOCs

The SKOV3, OVCAR3 human epithelial ovarian cancer (EOC) cell lines were purchased from ATCC (Rockville, MD, USA). The SKOV3 cells were cultured in DMEM/F12 HAM (Dulbecco's modified Eagle's medium; Sigma-Aldrich), supplemented with 10% foetal calf serum (FCS; PAA Laboratories, Somerset, UK), 100 μg/ml streptomycin, and 100 U/ml penicillin (Sigma-Aldrich) and aseptically grown at 37°C in a humidified incubator containing 5% CO_2_. The OVCAR3 cells were cultured in 1640 medium (Sigma-Aldrich), supplemented with 10% FCS, 100 μg/ml streptomycin, and 100 U/ml penicillin and aseptically grown at 37°C in a humidified incubator containing 5% CO_2_.

In order to generate spheroid, the SKOV3 and OVCAR3 cells were typsinised and cultured in 96-well ultra-low attachment (ULA) cluster plate (Greiner Bio-One, Germany) at a density of 2 × 10^4^ or 5 × 10^4^ cells/well, respectively and aseptically grown at 37°C in a humidified incubator containing 5% CO_2_. The half of medium (100 μl) was replaced by the fresh medium every two days. The sphere formation was maintained for 6 hours, 24 hours, 48 hours, 72 hours, 5 days and 7 days, respectively, subjected to sequential relevant experiments. Spheroid formation was continuously monitored in for one week using a Leica microscope (Leica CM1900) and Las EZ image capture software to take photographs. The cell density of each spheroid was quantified by an eyepiece systemic point-sampling grid as described previously [40], which contains 100 and 50 lines to count the number of points overly cell nuclei at 400 × mangification. Measurement were averaged over five microscopic fields to obtain an indexed percentage.

In order to assess the spheroid formation, the monolayer cells were dyed with CellTracker^TM^ Orange CMRA (1 μg/ml, 37°C, 20 minutes) (Life Technologies), and then trysinsied, followed by cultured in 96-well ULA plate at the density of 2 × 10^4^ (SKOV3) or 5 × 10^4^ (OVCAR3) cells/well. Spheroid formed was visualized using a confocal Leica microscopy and photographed using a Hamamatsu digital camera. The area of each spheroid was measured by Image-J software. Area fold was calculated by dividing the final area of spheroid at time 48 hours by of the spheroid at time 6 hours (Area Ratio = Spheroid Area_48h_ / Spheroid Area_6h_).

### Western blot analysis of cancer stem cell marker

Total cellular protein was isolated from monolayer and spheroid EOC cells. Briefly, the SKOV3 cells of each group were wash with ice-cold PBS twice and lysed in a buffer comprising 50 mM Tris-base, 5 mM EGTA, 150 mM NaCl, 1% Triton X-100, 100 μg/ml PMSF, 10 μg/ml aprotinin, 10 μg/ml leupeptin, 5 mM sodium vanadate and 50 mM sodium fluoride, then clarified by centrifugation (13000 rpm, 15 minutes, 4°C), and quantified by DC Protein Assay kit (Bio-Rad Laboratories, Hertfordshire, UK). Thirty μg of protein extract per lane were separated by SDS-PAGE using 10% gels. Proteins were transferred onto a PVDF membrane and blocked with 5% non-fat milk. Then PVDF membrane was incubated with respective primary antibodies directed against ALDH1A1 (1:2000) (Sigma-Aldrich) and GAPDH (1:3000) (Biosciences, San Diego, CA, USA) at 4°C overnight. Immunoreactive bands were visualized by incubating with HRP-conjugated mouse or rabbit anti-IgG (1:3000) (Santa Cruz Biotechnology, Inc., CA, USA) for 1 hour at RT, then followed by exposure to enhanced chemiluminescence reagent (Luminata chemiluminescence detection kit, Merck-Millipore).

### *In vitro* cytotoxicity (MTT) assay

10 μl of the MTT solution (Sigma-Aldrich) was added to each well, and the plates were incubated for 4 hours at 37°C. In monolayer cultures, the medium was removed and added 150 μl DMSO to dissolve the formazan crystals. In spheroid cultures, the formazan crystals were collected by centrifugation (13000 rpm, 10 minutes), dissolved in DMSO. The absorbance was quantified using a Bio-Tek Elx800 multi-plate reader (Bio-Tek Instruments Inc., VT, USA) at 540 nm.

### Drug treatments

Curcumin (Sigma-Aldrich) was dissolved in dimethylsulfoxide (DMSO) to make a 40 mM stock solution and was added directly to the media at different concentrations. Cells were treated with 0.3% DMSO as the negative control. The dosing of curcumin we chose in this study was based on previous reports [41, 42].

24 hours after seeding in 96-well plate as monolayer cultures or spheroids, the SKOV3 cells were treated with curcumin (0–100 μΜ for the monolayer culture and 0–200 μΜ for the spheroids,) cisplatin (0–100 μM for both monolayer culture and the spheroids) for 48 hours, subjected to the sequential relevant experiments.

In order to assess the combined effects of cisplatin and curcumin on the ovarian cancer spheroids, the responses were measured by Bliss independence reference models of synergy [43], using Graphpad Prism^®^ (Version 6.01, GraphPad Software, San Diego California USA, http://www.graphpad.com). The Bliss independence model is defined by the equation: Fab = Fa + Fb – Fa × Fa for 0 < F < 1. The fractional response to drug A alone at any dose is Fa. Similar the fractional response to drug B alone is Fb. Fab is the total response to a mixture of the two drugs. The combined effects of the two drugs as predicted by their individual effects Fa and Fb. The dose-response curve for drugs A and B administrated together is very close to the sum of the two individual dose-response curves, the combined effects are conforms to the additive model. In contrast, the combined effects are defined as synergistic model if the dose-response curve of Fab is larger than the sum of the two individual dose-response curves. In this case, Fa is the response curve for cisplatin while Fb is the response to curcumin. Fab would be the combined effects of cisplatin and curcumin.

### Adhesion assay

In preparation for the assay, a 96-well plate was pre-coated with 5 μg Matrigel (BD Matrigel matrix, Matrigel basement membrane matrix, Biosciences) diluted in DMEM/F12 HAM without FCS. The SKOV3 cell suspensions for monolayer and spheroid at density of 2 × 10^4^ cells/well in complete media were added into each well and left to adhere at 37°C, with 5% CO_2_ for 45 minutes. Then the wells washed with PBS to remove any unbound cells. The cells that had remained adhered were fixed with 4% formalin and stained with 0.5% crystal violet. The adherent cells were visualized under the microscope and counted by the image-J. The number of seeding cells was adjusted by the trypan blue (Seeding cells = 2 × 10^4^cells/cell viability rate).

### Invasion assay

In preparation for the assay, a 24-well Transwell insert with 8.0 μm pore size (Greiner Bio-One, Germany) was pre-coated with 50 μg Matrigel diluted in DMEM/F12 HAM without FCS. The SKOV3 cell suspensions for monolayer and spheroid (2 × 10^4^ cells/well in serum-free growth media + 0.1% FCS) were added to the upper compartment of the insert. Media containing a chemoattractant (10% FCS) was added to the bottom chamber of the Transwell plates. Following incubation at 37°C for 48 hours, non-invaded cells (which remained on the upper surface of the filter) were removed and invaded cells (on the lower surface of the filter) were stained with 0.5% crystal violet. The invaded cells were visualized under the microscope and counted by the image-J. The number of seeding cells was adjusted by the trypan blue (Seeding cells = 2 × 10^4^ cells/cell viability rate).

### Mesothelial clearance assay

The SKOV3 cells in monolayer were labelled with CellTracker^™^ Oregon Green 488 (1 μg/ml, 37°C, 20 minutes) (Life Technologies) and subjected to the sphere formation, followed by curcumin treatment (30 μM) for 48 hours. The LP-9 cells (A human-derived peritoneal mesothelial cell line) were purchased from ATCC (Rockville, MD, USA), and cultured in a 96-well plate with a ratio of Medium199 (Sigma-Aldrich) and Medium MCDB 105 (Sigma-Aldrich) supplemented with 10% FCS, 100 μg/ml streptomycin, and 100 U/ml penicillin to form confluent monolayers. The mesothelial cells labelled with CellTracker^™^ Orange CMRA (1 μg/ml, 37°C, 20 minutes), then the spheroids were transferred atop of the confluent monolayer of LP-9 gently (in 6 replicates per condition) and co-cultured for 24 hours. Each well was examined on an Olympus microscope and photographed using a Hamamatsu digital camera at the time points of 0 hour and 24 hours. The nonfluorescent area, created by the invading spheroid, in the CellTracker^TM^ Orange CMRA labelled mesothelial monolayer images were measured at 24 hours and divided by the initial area of the spheroid at time 0 (Clearance Area = Clearance Area_24h_ / Clearance Area_0h_).

### Statistical analysis

All experiments were at least repeated three times. All data were expressed as means ± SEM; Statistical analysis was performed on the variables in this study using either the Student's two-tailed *t*-test or one-way ANOVA. A *P*-value < 0.05 was considered to indicate a statistically significant result. Graphpad Prism^®^ (Version 4.03, GraphPad Software, San Diego California USA, http://www.graphpad.com) were used for data and graphic analysis.

## References

[1] Siegel R, Naishadham D, Jemal A (2013). Cancer statistics, 2013. CA Cancer J Clin.

[2] Coleman MP, Forman D, Bryant H, Butler J, Rachet B, Maringe C, Nur U, Tracey E, Coory M, Hatcher J, McGahan CE, Turner D, Marrett L (2011). Cancer survival in Australia, Canada, Denmark, Norway, Sweden, and the UK, 1995–2007 (the International Cancer Benchmarking Partnership): an analysis of population-based cancer registry data. Lancet (London, England).

[3] Cooke SL, Brenton JD (2011). Evolution of platinum resistance in high-grade serous ovarian cancer. Lancet Oncol.

[4] Steeg PS (2006). Tumor metastasis: mechanistic insights and clinical challenges. Nat Med.

[5] Lengyel E (2010). Ovarian cancer development and metastasis. Am J Pathol.

[6] Liao J, Qian F, Tchabo N, Mhawech-Fauceglia P, Beck A, Qian Z, Wang X, Huss WJ, Lele SB, Morrison CD, Odunsi K (2014). Ovarian cancer spheroid cells with stem cell-like properties contribute to tumor generation, metastasis and chemotherapy resistance through hypoxia-resistant metabolism. PLoS ONE.

[7] Sodek KL, Ringuette MJ, Brown TJ (2009). Compact spheroid formation by ovarian cancer cells is associated with contractile behavior and an invasive phenotype. Int J Cancer.

[8] Samardzija C, Luwor RB, Volchek M, Quinn MA, Findlay JK, Ahmed N (2015). A critical role of Oct4A in mediating metastasis and disease-free survival in a mouse model of ovarian cancer. Mol Cancer.

[9] Aggarwal BB, Deb L, Prasad S (2015). Curcumin differs from tetrahydrocurcumin for molecular targets, signaling pathways and cellular responses. Molecules (Basel, Switzerland).

[10] Zhao G, Han X, Zheng S, Li Z, Sha Y, Ni J, Sun Z, Qiao S, Song Z (2016). Curcumin induces autophagy, inhibits proliferation and invasion by downregulating AKT/mTOR signaling pathway in human melanoma cells. Oncol Rep.

[11] He M, Li Y, Zhang L, Li L, Shen Y, Lin L, Zheng W, Chen L, Bian X, Ng HK, Tang L (2014). Curcumin suppresses cell proliferation through inhibition of the Wnt/beta-catenin signaling pathway in medulloblastoma. Oncol Rep.

[12] Prasad CP, Rath G, Mathur S, Bhatnagar D, Ralhan R (2009). Potent growth suppressive activity of curcumin in human breast cancer cells: Modulation of Wnt/beta-catenin signaling. Chem Biol Interact.

[13] Shehzad A, Lee J, Huh TL, Lee YS (2013). Curcumin induces apoptosis in human colorectal carcinoma (HCT-15) cells by regulating expression of Prp4 and p53. Mol Cell.

[14] Li Y, VandenBoom TG, Kong D, Wang Z, Ali S, Philip PA, Sarkar FH (2009). Up-regulation of miR-200 and let-7 by natural agents leads to the reversal of epithelial-to-mesenchymal transition in gemcitabine-resistant pancreatic cancer cells. Cancer Res.

[15] Watson JL, Greenshields A, Hill R, Hilchie A, Lee PW, Giacomantonio CA, Hoskin DW (2010). Curcumin-induced apoptosis in ovarian carcinoma cells is p53-independent and involves p38 mitogen-activated protein kinase activation and downregulation of Bcl-2 and survivin expression and Akt signaling. Mol Carcinog.

[16] Hossain DM, Bhattacharyya S, Das T, Sa G (2012). Curcumin: the multi-targeted therapy for cancer regression. Front Biosci (Scholar edition).

[17] Olivera A, Moore TW, Hu F, Brown AP, Sun A, Liotta DC, Snyder JP, Yoon Y, Shim H, Marcus AI, Miller AH, Pace TW (2012). Inhibition of the NF-kappaB signaling pathway by the curcumin analog, 3,5-Bis(2-pyridinylmethylidene)-4-piperidone (EF31): anti-inflammatory and anti-cancer properties. Int Immunopharmacol.

[18] Johnson SM, Gulhati P, Arrieta I, Wang X, Uchida T, Gao T, Evers BM (2009). Curcumin inhibits proliferation of colorectal carcinoma by modulating Akt/mTOR signaling. Anticancer Res.

[19] Dandawate P, Padhye S, Ahmad A, Sarkar FH (2013). Novel strategies targeting cancer stem cells through phytochemicals and their analogs. Drug Deliv Transl Res.

[20] Jaiswal AS, Marlow BP, Gupta N, Narayan S (2002). Beta-catenin-mediated transactivation and cell-cell adhesion pathways are important in curcumin (diferuylmethane)-induced growth arrest and apoptosis in colon cancer cells. Oncogene.

[21] Elamin MH, Shinwari Z, Hendrayani SF, Al-Hindi H, Al-Shail E, Khafaga Y, Al-Kofide A, Aboussekhra A (2010). Curcumin inhibits the Sonic Hedgehog signaling pathway and triggers apoptosis in medulloblastoma cells. Mol Carcinog.

[22] Wang Z, Zhang Y, Banerjee S, Li Y, Sarkar FH (2006). Notch-1 down-regulation by curcumin is associated with the inhibition of cell growth and the induction of apoptosis in pancreatic cancer cells. Cancer.

[23] Gupta SC, Patchva S, Aggarwal BB (2013). Therapeutic roles of curcumin: lessons learned from clinical trials. AAPS J.

[24] Pastrana E, Silva-Vargas V, Doetsch F (2011). Eyes wide open: a critical review of sphere-formation as an assay for stem cells. Cell Stem Cell.

[25] James MI, Howells LM, Karmokar A, Higgins JA, Greaves P, Cai H, Dennison A, Metcalfe M, Garcea G, Lloyd DM, Berry DP, Steward WP, Brown K (2015). Characterization and propagation of tumor initiating cells derived from colorectal liver metastases: trials, tribulations and a cautionary note. PloS ONE.

[26] Luo X, Dong Z, Chen Y, Yang L, Lai D (2013). Enrichment of ovarian cancer stem-like cells is associated with epithelial to mesenchymal transition through an miRNA-activated AKT pathway. Cell Prolif.

[27] Condello S, Morgan CA, Nagdas S, Cao L, Turek J, Hurley TD, Matei D (2015). beta-Catenin-regulated ALDH1A1 is a target in ovarian cancer spheroids. Oncogene.

[28] Lawrenson K, Benjamin E, Turmaine M, Jacobs I, Gayther S, Dafou D (2009). In vitro three-dimensional modelling of human ovarian surface epithelial cells. Cell Prolif.

[29] Chen K, Huang YH, Chen JL (2013). Understanding and targeting cancer stem cells: therapeutic implications and challenges. Acta Pharmacol Sin.

[30] Kipps E, Tan DS, Kaye SB (2013). Meeting the challenge of ascites in ovarian cancer: new avenues for therapy and research. Nat Rev.

[31] Naora H, Montell DJ (2005). Ovarian cancer metastasis: integrating insights from disparate model organisms. Nat Rev.

[32] Lowe KA, Chia VM, Taylor A, O’Malley C, Kelsh M, Mohamed M, Mowat FS, Goff B (2013). An international assessment of ovarian cancer incidence and mortality. Gynecol Oncol.

[33] Weiswald LB, Bellet D, Dangles-Marie V (2015). Spherical cancer models in tumor biology. Neoplasia.

[34] Luvero D, Milani A, Ledermann JA (2014). Treatment options in recurrent ovarian cancer: latest evidence and clinical potential. Ther Adv Med Oncol.

[35] Vaughan S, Coward JI, Bast RC, Berchuck A, Berek JS, Brenton JD, Coukos G, Crum CC, Drapkin R, Etemadmoghadam D, Friedlander M, Gabra H (2011). Rethinking ovarian cancer: recommendations for improving outcomes. Nat Rev.

[36] Banerjee S, Kaye SB (2013). New strategies in the treatment of ovarian cancer: current clinical perspectives and future potential. Clin Cancer Res.

[37] Shehzad A, Lee YS (2013). Molecular mechanisms of curcumin action: signal transduction. Biofactors.

[38] Yallapu MM, Maher DM, Sundram V, Bell MC, Jaggi M, Chauhan SC (2010). Curcumin induces chemo/radio-sensitization in ovarian cancer cells and curcumin nanoparticles inhibit ovarian cancer cell growth. J Ovarian Res.

[39] Garige M, Walters E (2015). Curcumin inhibits development and cell adhesion in Dictyostelium discoideum: Implications for YakA signaling and GST enzyme function. Biochem Biophys Res Commun.

[40] Parra ER, Bielecki LC, Ribeiro JM, Andrade Balsalobre F, Teodoro WR, Capelozzi VL (2010). Association between decreases in type V collagen and apoptosis in mouse lung chemical carcinogenesis: a preliminary model to study cancer cell behavior. Clinics (Sao Paulo).

[41] Hatcher H, Planalp R, Cho J, Torti FM, Torti SV (2008). Curcumin: from ancient medicine to current clinical trials. Cell Mol Life Sci.

[42] Tsai JR, Liu PL, Chen YH, Chou SH, Cheng YJ, Hwang JJ, Chong IW (2015). Curcumin Inhibits Non-Small Cell Lung Cancer Cells Metastasis through the Adiponectin/NF-kappab/MMPs Signaling Pathway. PLoS ONE.

[43] Zhao W, Sachsenmeier K, Zhang L, Sult E, Hollingsworth RE, Yang H (2014). A New Bliss Independence Model to Analyze Drug Combination Data. J Biomol Screen.

